# Strong Interactions between Austenite and the Matrix of Medium-Mn Steel during Intercritical Annealing

**DOI:** 10.3390/ma13153366

**Published:** 2020-07-29

**Authors:** Tianpeng Zhou, Cunyu Wang, Chang Wang, Wenquan Cao, Zejun Chen

**Affiliations:** 1 College of Materials Science and Engineering, Chongqing University, Chongqing 400044, China; ztp@cqu.edu.cn; 2 Central Iron & Steel Research Institute, Haidian, Beijing 100081, China; wangcunyu@nercast.com (C.W.); wangchang@nercast.com (C.W.)

**Keywords:** austenite nucleation, matrix recrystallization, strong interaction, Kurdjumov Sachs orientation relationship

## Abstract

The effects of heat treatment on the microstructure evolution was studied in regards to austenite nucleation and grain growth. It was found that the austenite nucleation and matrix recrystallization kinetics of samples annealed at 675 °C for different times were revealed, implying a strong interaction between the ferrite matrix and austenite. The recrystallization of the matrix during annealing provided favorable conditions for austenite nucleation and growth, and the formation of austenite during this process reduced the matrix recrystallization kinetics, thus delaying the recrystallization process of the matrix around the austenite grains. The statistical results for the austenite grain size under different annealing temperatures indicated that the average grain size of the austenite slightly increases with increasing of the annealing temperature, but the austenite with the largest grain size grows faster at the same temperature. This difference is attributed to the strict Kurdjumov Sachs (KS) orientation relationship (OR) between the austenite grains and the matrix, because the growth of austenite with a strict KS OR with the matrix is often inhibited during annealing. In contrast, the austenite maintains a non-strict KS OR with the matrix and can grow preferentially with increasing annealing temperature and time.

## 1. Introduction

The development of advanced ultrahigh strength steels is promoted by the need for lightweight bodies, and medium-Mn steels with 3–10% Mn are considered one of the most promising materials for automobile mass reduction [1,2,3]. When medium-Mn steels were first developed by Miller in 1972 [4], a substantial amount of research and development work had been carried out. During this period, Professor Morris studied high-toughness steels in the 1980s and found that a large number of austenite and ultrafine ferrite structures could be obtained in low-carbon steel with a 5% Mn mass fraction by austenite reverse transformation [5]. Currently, medium-Mn steels have attracted an essential attention in the research and development of automobile steels due to their mixed structural characteristics and excellent mechanical properties.

The mechanical properties of medium-Mn steels are closely related to the microstructure, which is affected by the annealing conditions. A typical heat treatment process during the production of cold-rolled medium-Mn steels produces a mixed structure of ferrite (F) and retained austenite (A) by an intercritical annealing process [6,7,8,9,10]. The cold-rolled medium-Mn steel was heat treated between Ac1 and the Ac3 temperature, which was called intercritical annealing. In the intercritical annealing process, some austenite would develop by means of austenite reversion, which resulted in a duplex microstructure of both reverted austenite phase and the annealed martensite phase [9,10]. However, the microstructure evolution of cold-rolled medium-Mn steels during intercritical annealing is a complex process that involves the recovery and recrystallization of the matrix and the formation and growth of austenite. Especially the research on retained austenite in steel, which is not only related to the mechanical properties of materials, but also widely studied in the complex postprocessing of automobile parts [11].

There are reports about a potential relationship between matrix recrystallization and phase transformation processes during the intercritical annealing process of cold-rolled steels. Based on a steel with the composition of Fe-1.48Mn-0.013Si-0.15C, Chbihi revealed interactions between ferrite recrystallization and austenite formation in high-strength steels [12]. Yang observed the relationship between austenite nucleation and matrix recrystallization in a 0.08C-1.45Mn-0.21Si steel [13]. Zheng found that not only the transformation kinetics but also the morphology and spatial distribution of austenite were affected by matrix recrystallization in a cold-rolled dual-phase steel during intercritical annealing [14]. Although there are descriptions about the microstructure evolution of medium-Mn steels during the intercritical annealing process, the interactions between the matrix and austenite during the microstructure evolution process are still ambiguous.

The primary objective herein is to study the recrystallization behavior of the matrix and austenite nucleation and growth in medium-Mn steel under the synergistic influences of the annealing temperature and time to reveal their intrinsic relationship.

## 2. Experimental Procedures

A steel ingot with a chemical composition of Fe-0.15C-5.2Mn-1.1Al-0.1Si -0.05Cr (in percent mass) was prepared by vacuum induction melting. After solution treatment at 1200 °C for 2 h, a 30-mm-thick plate was hot rolled to produce 4-mm-thick sheets. The rolling temperature is 1000 °C, the finishing temperature is controlled at 800 °C. After soft annealing at 650 °C for 6 h, the hot-rolled plate was cold-rolled to form a 1.6-mm-thick sheet at room temperature. The critical annealing temperatures of Ac1 (640 °C) and Ac3 (800 °C) of the studied steel were calculated using Thermo-Calc software, and intercritical annealing was carried out within the dual phase (α + γ) region with temperatures range of 650–725 °C and different times.

The microstructure of the hot-rolled, cold-rolled and annealed specimens were observed using field-emission scanning electron microscopy (SEM, TESCAN VEGA 3). Samples for XRD and EBSD were mechanically ground and finally electropolished in a mixture of 8% perchloric acid and 92% ethanol (vol.%) at −20 °C with a potential of 30 V for 30 s to remove the layers damaged by mechanical polishing. Measurements of the volume fraction of retained austenite was conducted by Rigaku DMAX 2500 PC X-ray diffractometer using a Cu *K*α_1_ radiation source (*λ* = 1.5405 Å). Samples were scanned over a 2*θ* range from 40° to 100° with a step size of 0.02°. Ferrite plane diffraction lines of {200}, {211} and austenite plane diffraction lines of {200}, {220}, {311} were measured for the calculation of the volume fraction of austenite according to Equation (1) [15].

(1) $$\begin{array}{c} V_{A}=\frac{1}{6}(\frac{1}{1+2.5\cdot \frac{I_{F\left(200\right)}}{IA_{\left(200\right)}}}+\frac{1}{1+1.38\cdot \frac{I_{F\left(200\right)}}{I_{A\left(220\right)}}}+\frac{1}{1+2.02\cdot \frac{I_{F\left(200\right)}}{I_{A\left(311\right)}}}+\frac{1}{1+1.19\cdot \frac{I_{F\left(211\right)}}{I_{A\left(200\right)}}}\\ +\frac{1}{1+0.06\cdot \frac{I_{F\left(211\right)}}{I_{A\left(220\right)}}}+\frac{1}{1+0.96\cdot \frac{I_{F\left(211\right)}}{I_{A\left(311\right)}}}) \end{array}$$

where $V_{A}$ is the volume fraction of austenite, $I_{A}$ and $I_{F}$ are the integrated intensity of austenite and ferrite plane diffraction lines. *G* is the ratio of the intensity related factors corresponding to the austenite plane and the ferrite plane. *G*-value for each $I_{F}/I_{A}$ was used as follow, 2.5 for $I_{F\left(200\right)}/I_{A\left(200\right)}$, 1.38 for $I_{F\left(200\right)}/I_{A\left(220\right)}$, 2.02 for $I_{F\left(200\right)}/I_{A\left(311\right)}$, 1.19 for $I_{F\left(211\right)}/I_{A\left(200\right)}$, 0.06 for $I_{F\left(211\right)}/I_{A\left(220\right)}$, 0.96 for $I_{F\left(211\right)}/I_{A\left(311\right)}$.

## 3. Results

### 3.1. Microstructure Change before Intercritical Annealing

**Figure 1:**
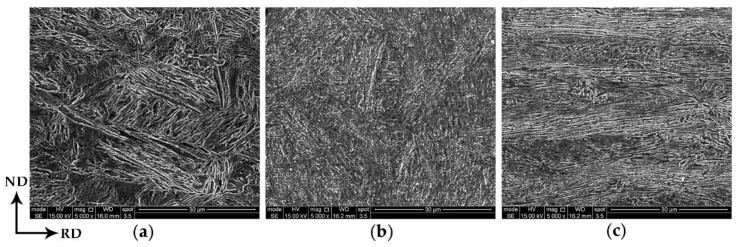
Microstructure of the (**a**) hot-rolled, (**b**) soft-annealed and (**c**) cold-rolled samples. The images are taken in the section including rolling direction (RD) and normal direction (ND) as indicated in this figure.

In the present investigation, the hot-rolled steel showed a fully martensitic microstructure (Figure 1a), which showed clearly the martensitic microstructure and assumed very high hardness. For further cold rolling, the hot rolled steel had to be annealed to reduce the hardness, which was called soft annealing. Figure 1b shows the SEM image of the steel after soft annealing at 650 °C for 6 h. It can be seen that the martensitic microstructure changed into recovered martensitic microstructure with some carbides developed during the soft annealing. Figure 1c shows the cold rolled microstructure, it can be seen that most of the recovered martensitic lath were rotated into the rolling direction and others were featured with zigzag shape, resulting from the heavy rolling.

### 3.2. Microstructure of the Samples Annealed for Different Times

The annealed specimens prepared from the cold-rolled plate were heated to 675 °C at a heating rate of approximately 10 °C/s and then isothermally annealed from 1 to 360 min, followed by cooling to room temperature by air.

**Figure 2:**
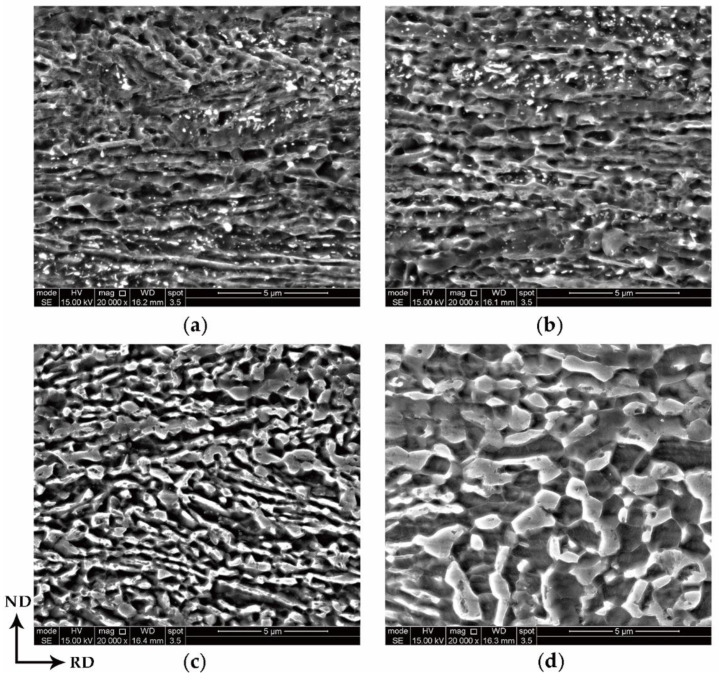
SEM micrographs of the samples intercritically annealed at 675 °C for different times: (**a**) 1 min, (**b**) 3 min, (**c**) 30 min and (**d**) 360 min.

Samples annealed at 675 °C for different times are presented in Figure 2a–d. The amount of carbide precipitation gradually decreased and disappeared completely within 30 min during intercritical annealing. Morphologically, it was difficult to distinguish the fine austenite mixed with the ferrite matrix after annealing for a short time. With increasing annealing time, the austenite and ferrite grains coarsened, and were easier to distinguish. The matrix changed from ferritic lath microstructure to a well-defined and equiaxed microstructure, which indicated that the matrix recovered and recrystallized.

**Figure 3:**
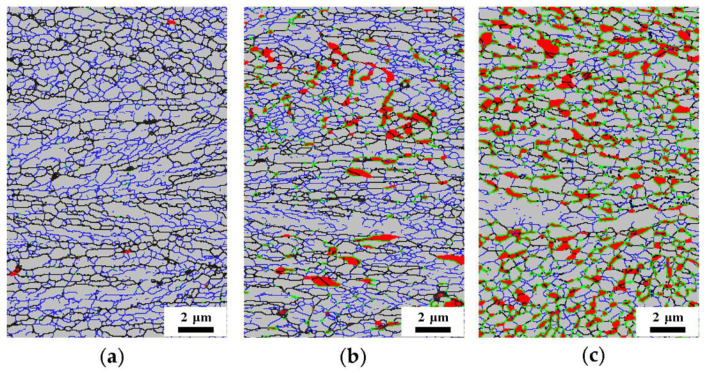

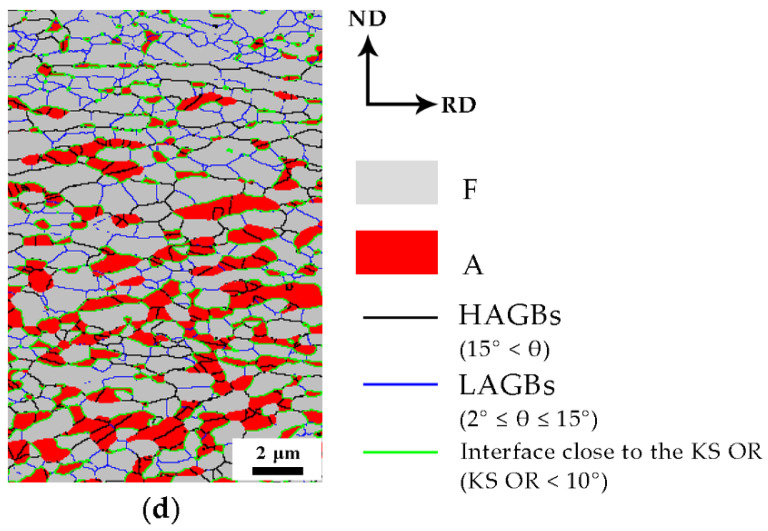
EBSD micrographs of the samples intercritically annealed at 675 °C for different times: (**a**) 1 min, (**b**) 3 min, (**c**) 30 min and (**d**) 360 min.

The EBSD micrographs of the samples in Figure 3a–d show the microstructure evolution of the cold-rolled steel during annealing at 675 °C for different times. When the isothermal time increased from 1 to 360 min, the main changes included an increase in the austenite volume fraction and grain size. Obviously, the recrystallized ferrite grains exhibited a granular shape as shown in Figure 3. These results are consistent with the SEM results.

In addition, it can be seen that the low-angle $\left(2^{{}^{\circ}}\leq \theta \leq 15^{{}^{\circ}}\right)$ grain boundaries (LAGBs) decreased obviously. It was also observed that almost all retained austenite grains were usually distributed along the high-angle $\left(15^{{}^{\circ}}<\theta \right)$ grain boundaries (HAGBs) of the ferrite matrix, and a typical Kurdjumov Sachs (KS) orientation relationship (OR), viz. ${\left[10\bar{1}\right]}_{\gamma }\left|\left|{\left[11\bar{1}\right]}_{\alpha }\;\mathrm{and}\;{\left(111\right)}_{\gamma }\right|\right|{\left(011\right)}_{\alpha }$, was found between the austenite grains and adjacent ferrite grains [16], as marked by the green lines.

### 3.3. Microstructure of the Samples Annealed at Different Temperatures

**Figure 4:**
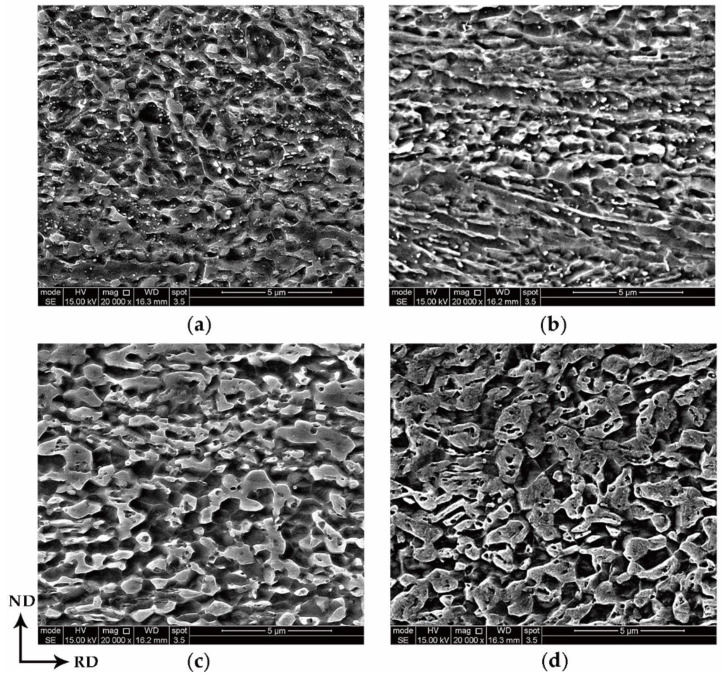
Microstructure of the samples: (**a**) A650, (**b**) A675, (**c**) A700 and (**d**) A725.

To reveal the relationship between the microstructure evolution and intercritical annealing temperature, the cold-rolled samples were annealed at 650, 675, 700 and 725 °C for 10 min and are referred to as A650, A675, A700 and A725, respectively. A detailed crystallographic analysis of the microstructure of the A650, A675, A700, and A725 samples was performed by SEM, as shown in Figure 4.

In all the samples, carbides only appeared in samples A650 and A675 and decreased rapidly with increasing temperature. At the same time, the grains coarsened with increasing annealing temperature.

**Figure 5:**
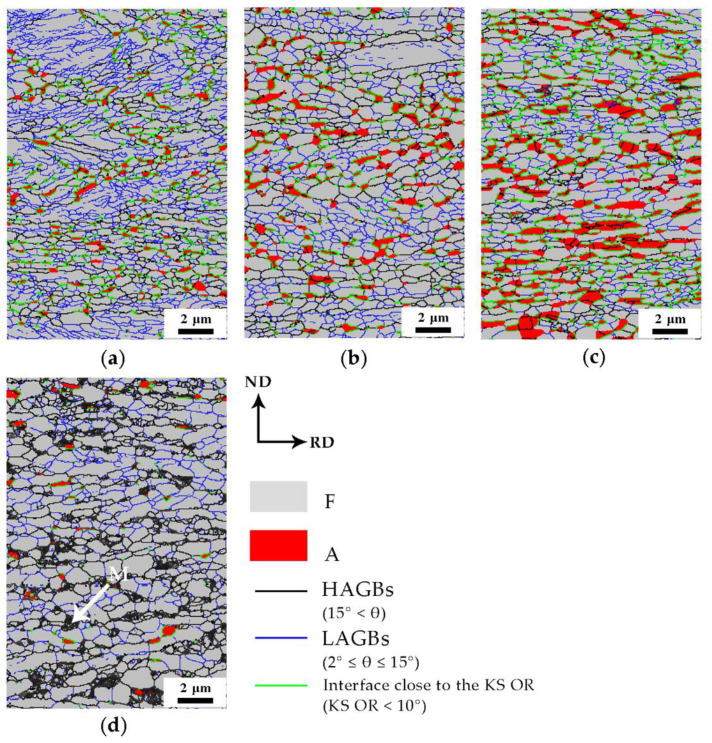
EBSD micrographs of samples (**a**) A650, (**b**) A675, (**c**) A700 and (**d**) A725.

Based on the microstructure in Figure 5, an obvious growth of austenite was observed with an increase in the annealing temperature. It is worth noting that when the temperature reached 725 °C, a large number of austenite grains transformed into martensite (M) during the cooling process (Figure 5d), which was caused by an increase in austenite grain size and a decrease in the element content in austenite [17,18].

## 4. Discussion

### 4.1. Ferrite Recrystallization and Formation of Austenite

From a statistical point of view, it is interesting that a large number of austenite grains were distributed at the high-angle grain boundaries in the matrix, but there were still austenite grains distributed at the low-angle grain boundaries, although they were small in number and size. Therefore, we believe that different grain boundary types were not one of the main factors that impacted the nucleation and growth of the austenite.

**Figure 6:**
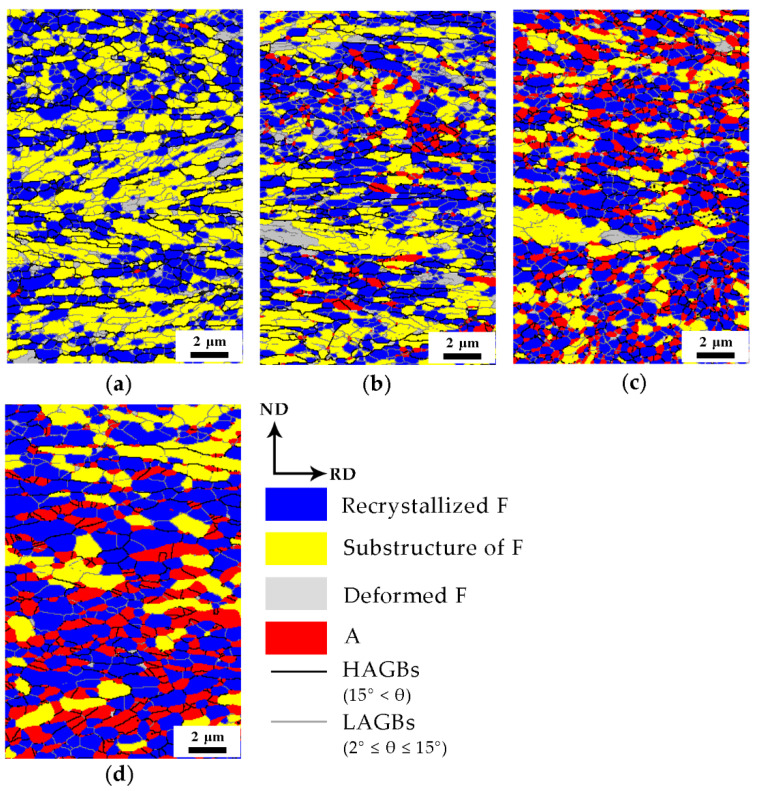
The evolution of the matrix microstructure for different intercritical annealing times at 675 °C of (**a**) 1 min, (**b**) 3 min, (**c**) 30 min and (**d**) 360 min.

Studies have shown that defects (e.g., dislocations and grain boundaries) in the matrix can act as diffusion channels, accelerate element diffusion, and influence austenite nucleation and growth [12,19,20]. The recovery and recrystallization of cold-rolled medium-Mn steel during the annealing process could change these defect conditions, so it is necessary to study the microstructure evolution of the matrix after annealing. The recrystallized fractions for different annealing times were calculated from the EBSD data employing the grain orientation spread (GOS) approach [21,22,23]. Generally, based on the distribution statistics for a grain orientation, a GOS of 1–2° is the criterion to define the recrystallization grains in the software of HKL [21,22,23]. In this experiment, the GOS value of 1° was used to distinguish the recrystallized grains in the matrix. As shown in Figure 6, the recovery and recrystallization of matrix in the cold-rolled medium-Mn steel after different annealing times were analyzed. The austenite grains were clearly around the recrystallized ferrite matrix, so it was inferred that the formation of austenite was affected by the recrystallization state of the matrix. The recrystallization process provided favorable conditions for austenite nucleation and growth by refining the grains, introducing additional grain boundaries and accelerating element diffusion. Therefore, it is more advantageous for nucleation and growth of austenite grains around the recrystallized ferrite.

Obviously, the unrecrystallized ferrite (e.g., substructure of ferrite and deformed ferrite) did not readily provide additional grain boundaries due to the coarse grains. Therefore, it was difficult for austenite to nucleate in the unrecrystallized ferrite matrix.

In addition, upon considering the data in Figure 3a and Figure 6a, it was confirmed that a large amount of recrystallized ferrite occurred in the matrix of the cold-rolled medium-Mn steel after annealing at 675 °C for 1 min. At the same time, there was a small amount of austenite grains in the matrix, and austenite grains remained close to the KS OR with the surrounding recrystallized ferrite grains. It was reasonable to infer that the deformed ferrite partially recrystallized before austenite nucleation.

**Figure 7:**
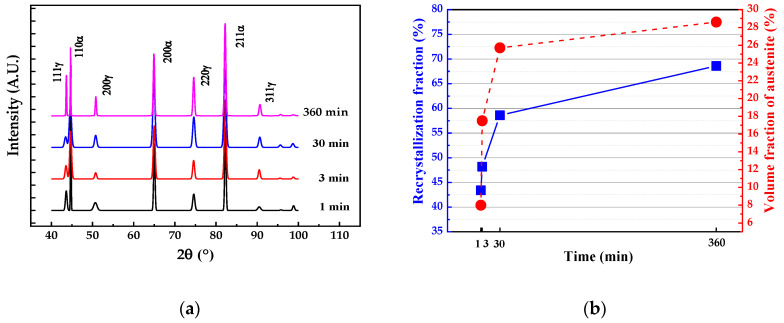
(**a**) XRD diffraction patterns and (**b**) the changes in the austenite volume fraction and recrystallization of the matrix with intercritical annealing time.

Based on the EBSD data employing the grain orientation spread (GOS) approach [21,22,23] for recrystallization fraction and Equation (1) [15] for austenite fraction calculation as mentioned above. The matrix recrystallization fraction and austenite volume fraction in the samples with different annealing times were calculated, and the results are shown in Figure 7. As the annealing time increased, both the recrystallization fraction of the matrix and the volume fraction of the austenite increased, which indicated that austenite nucleation and matrix recrystallization occurred simultaneously. Moreover, the recrystallization fraction and austenite volume fraction had a similar growth trend. It can be concluded that there was a certain relationship between them during the annealing process.

**Figure 8:**
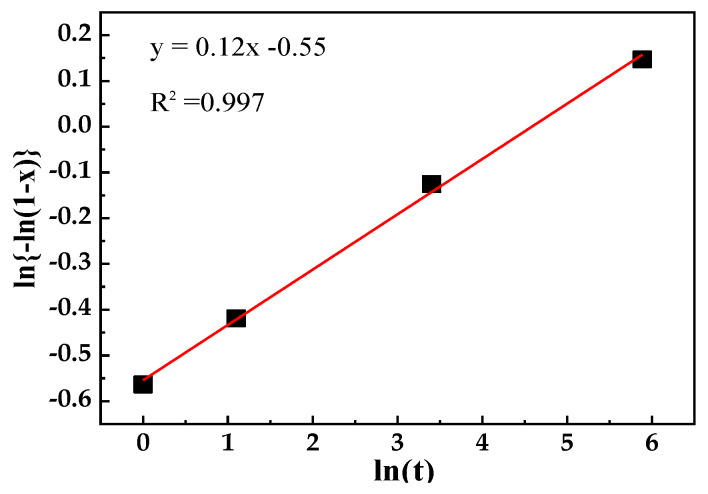
The ln{−ln(1 − *x*)} vs. ln(*t*) curve to determine the Avrami exponent of the specimens annealed at 675 °C for different times.

The classical Johnson Mehl Avrami Kolmogorov (JMAK) model [24] was used to study the recrystallization kinetics of samples annealed at different times, as given in Equation (2):

(2) $$X=1-\exp\left(-kt^{n}\right)$$

where X is the recrystallized fraction of ferrite matrix and *k*, *t*, and *n* are the reaction constant, annealing time and Avrami exponent, respectively. The value of n can be expressed as the slope of the ln(−ln(1 − *X*)) vs. ln(*t*) plot (Figure 8), which characterizes the recrystallization kinetics of materials. When *n* is less than 1, it indicates that the recrystallization process is slow. In this experiment, the *n* value was only 0.12, which indicates that the presence of austenite inhibited the continuous recrystallization process, thus delaying the overall kinetics and the occurrence of recrystallization. It can also be seen in Figure 6 that there were still a large number of substructures due to the formation of austenite hindering the recrystallization of the surrounding matrix microstructure, resulting in a low Avrami exponent *n*.

In conclusion, there was a strong interaction between the formation of austenite and the recrystallization of the matrix. The promotion of the recrystallization process accelerated the nucleation and growth of the austenite, and the presence of austenite restricted the recrystallization process. Herein, the rate of the increase in the recrystallization fraction of ferrite matrix and the volume fraction of austenite decreased with an increase in the annealing time.

### 4.2. Factors that Influenced the Austenite Grain Size

**Figure 9:**
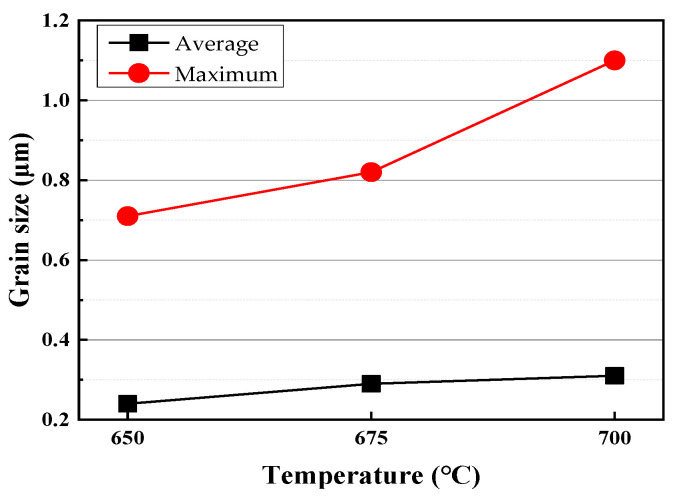
Changes in the average grain size and maximum grain size of austenite grains with annealing temperature.

The results showed that the austenite grain size increased with increasing annealing time and temperature. The grain size was calculated based on the boundary definition with misorientation no less than 10 degrees. The maximum grain size and average grain size of the austenite at different annealing temperatures were evaluated from EBSD maps, both of them are shown in Figure 9. It can be seen that no significant difference with varied annealing temperature could be found for the average grain size, but the maximum grain size difference increased with increasing annealing temperature, which reflected the different controlling factors of the austenite grain growth.

In order to understand the different grain growth behavior, the microstructure under different annealing conditions were analyzed in details and several features could be as follows:

1. With increasing annealing time and temperature, the maximum grain size of austenite grains increases significantly.
2. The austenite grain size at low-angle grain boundaries was always small.
3. The austenite grain size was relatively uniform under low-temperature (650 °C) annealing conditions.

**Figure 10:**
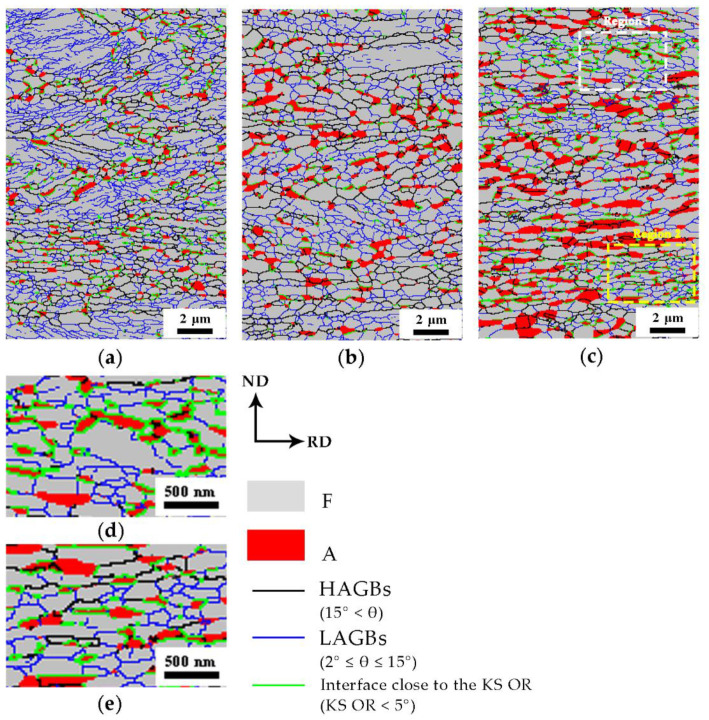
EBSD micrographs of samples (**a**) A650, (**b**) A675, (**c**) A700, (**d**) region 1 in (**c**), and (**e**) region 2 in (**c**).

Based on the EBSD analysis as shown in Figure 10, the austenite could nucleate both in the high angle boundary and the low angle boundary, which means that boundary misorientation does not affect the nucleation of the austenite grains. However, according to the statistical results, it seems that the growth of the austenite at the low-angle grain boundaries was limited. Therefore, the diffusion of elements was not the only factor affecting the growth of the austenite as discussed in Section 4.1. It is believed that there was another important factor controlling the growth of the austenite grains.

According to Equation (3) [25]:

(3) $$\nu =M\cdot \Delta G/V_{m}$$

where ν, and M are interface velocity and interface mobility of A/F interface, respectively. ΔG is the free energy dissipation at the interface for 1 mol of the substitutional atoms transferred across the interface, and $V_{m}$ is the molar volume. It is generally considered that an interface with good coherency has a lower intrinsic interface mobility. The interface between the austenite and the ferrite matrix with a KS OR had an improved coherency, so it had a decreased interface mobility. In short, under the same conditions, the growth of austenite grains with a strict KS OR with the ferrite matrix was limited due to the decreased interface velocity.

In order to understand the effects of KS OR on the grain growth of the austenite, the KS OR between austenite and the ferrite was limited to 5°. It was revealed by the analysis of the EBSD map that the austenite grains with large grain sizes gradually broke away from the KS OR with the ferrite matrix, while the austenite grains with a strict KS OR were small, which proves that the KS OR did have a retarding effect on the growth of the austenite grains.

Furthermore, the regions with mainly low angle boundary as shown in Figure 10c are enlarged and the detailed microstructure were revealed in Figure 10d and e. It can be seen that almost all austenite grains at low-angle grain boundaries still maintained a strict KS OR with the ferrite matrix, which inhibited the growth of these austenite grains and thus resulted in a relative small grain size developed from the low angle boundaries.

Apart from the influence of element diffusion and the KS OR, another important factor should be considered in this experiment. It is noteworthy that after annealing at 650 °C for 10 min, there were still carbides in the matrix. Research by Benzing showed that the presence of carbides retards the recrystallization of the matrix [26], and Gouné noted that carbides reduce the nucleation and growth kinetics of austenite [27]. Therefore, regardless of whether there was a strict KS OR between the austenite grains and ferrite matrix in the A650 sample, the austenite grains could not grow rapidly. The existence of carbides reduced the growth kinetics of all austenite grains. In contrast, with an increase in the annealing temperature, the dissolution of the carbides was accelerated, and the austenite grains that were in a favorable position for diffusion and had a non-strict KS OR with the ferrite matrix grew rapidly.

## 5. Conclusions

- During the annealing process of the cold-rolled medium-Mn steel, the recrystallization of the ferrite matrix provided favorable conditions for the nucleation and growth of the austenite, while the presence of austenite delayed the overall kinetics of recrystallization, thus inhibiting the continuous recrystallization process.
- The high- and low-angle grain boundaries in the matrix provided sites for austenite nucleation, which was related to the recrystallization of the ferrite matrix. However, austenite grains formed at the low-angle grain boundaries always maintained a strict KS OR with the ferrite matrix, so the growth of the austenite was restrained, and the grain size was decreased during intercritical annealing.
- The existence of carbides reduced the kinetics of the austenite growth, so it was difficult for the austenite to grow at low annealing temperatures, regardless of whether there was a strict KS OR between it and the matrix. With increasing annealing temperature, the austenite grains had a non-strict KS OR with the ferrite matrix and grew rapidly.
